# *Yersinia pestis* Interacts With SIGNR1 (CD209b) for Promoting Host Dissemination and Infection

**DOI:** 10.3389/fimmu.2019.00096

**Published:** 2019-03-12

**Authors:** Kun Yang, Yingxia He, Chae Gyu Park, Young Sun Kang, Pei Zhang, Yanping Han, Yujun Cui, Silvia Bulgheresi, Andrey P. Anisimov, Svetlana V. Dentovskaya, Xiaoling Ying, Lingyu Jiang, Honghui Ding, Olivia Adhiambo Njiri, Shusheng Zhang, Guoxing Zheng, Lianxu Xia, Biao Kan, Xin Wang, Huaiqi Jing, Meiying Yan, Wei Li, Yuanzhi Wang, Xiding Xiamu, Gang Chen, Ding Ma, Sara Schesser Bartra, Gregory V. Plano, John D. Klena, Ruifu Yang, Mikael Skurnik, Tie Chen

**Affiliations:** ^1^Department of Clinical Immunology, Tongji Hospital, Tongji Medical College, Huazhong University of Science and Technology, Wuhan, China; ^2^Department of Pathogen Biology and Immunology, Shihezi University School of Medicine, Shihezi, China; ^3^Laboratory of Immunology, Brain Korea 21 PLUS Project for Medical Science, Severance Biomedical Science Institute, Yonsei University College of Medicine, Seoul, South Korea; ^4^Department of Biomedical Sciences, College of Medicine, University of Illinois at Chicago, Chicago, IL, United States; ^5^State Key Laboratory of Pathogen and Biosecurity, Beijing Institute of Microbiology and Epidemiology, Beijing, China; ^6^Department of Ecogenomics and Systems Biology, University of Vienna, Vienna, Austria; ^7^State Research Center for Applied Microbiology and Biotechnology, Obolensk, Russia; ^8^Department of Biological Sciences, Faculty of Science, Technology and Engineering, Chuka University, Chuka, Kenya; ^9^National Institute for Communicable Diseases Control and Prevention, Chinese Center for Disease Control and Prevention, Beijing, China; ^10^Institute of Organ Transplantation, Tongji Hospital, Tongji Medical College, Huazhong University of Science and Technology, Wuhan, China; ^11^Department of Obstetrics and Gynecology, Tongji Hospital, Tongji Medical College, Huazhong University of Science and Technology, Wuhan, China; ^12^Department of Microbiology and Immunology, University of Miami Miller School of Medicine, Miami, FL, United States; ^13^School of Biological Sciences, University of Canterbury, Christchurch, New Zealand; ^14^Department of Bacteriology and Immunology, Haartman Institute, Helsinki University Central Hospital Laboratory Diagnostics, University of Helsinki, Helsinki, Finland

**Keywords:** *Yersinia pestis*, SIGNR1 (CD209b), macrophages, dendritic cells (DCs), antigen presenting cells (APCs), core lipopolysaccharide/lipooligosaccharides (core LPS/LOS), bacterial dissemination, host-pathogen interactions

## Abstract

*Yersinia pestis*, a Gram-negative bacterium and the etiologic agent of plague, has evolved from *Yersinia pseudotuberculosis*, a cause of a mild enteric disease. However, the molecular and biological mechanisms of how *Y. pseudotuberculosis* evolved to such a remarkably virulent pathogen, *Y. pestis*, are not clear. The ability to initiate a rapid bacterial dissemination is a characteristic hallmark of *Y. pestis* infection. A distinguishing characteristic between the two *Yersinia* species is that *Y. pseudotuberculosis* strains possess an O-antigen of lipopolysaccharide (LPS) while *Y. pestis* has lost the O-antigen during evolution and therefore exposes its core LPS. In this study, we showed that *Y. pestis* utilizes its core LPS to interact with SIGNR1 (CD209b), a C-type lectin receptor on antigen presenting cells (APCs), leading to bacterial dissemination to lymph nodes, spleen and liver, and the initiation of a systemic infection. We therefore propose that the loss of O-antigen represents a critical step in the evolution of *Y. pseudotuberculosis* into *Y. pestis* in terms of hijacking APCs, promoting bacterial dissemination and causing the plague.

## Introduction

*Yersinia pestis* is the bacterium that causes bubonic, septicemic, and pneumonic forms of plague and that was the cause of the Black Death in Europe during the middle ages. Recent studies have proved that all three suspected plague pandemics (the Justinian, the Black Death and the third pandemic) were caused by this bacterium (1–6). Based on a study (7), the New York Times on October 31, 2010 reported that the plague pathogen responsible for all known plague pandemics in the recorded history of human civilization might have originated in China, but more likely from Eurasia (8, 9). *Y. pesti*s directly evolved from *Y. pseudotuberculosis*, the cause of a self-limited mesenteric lymphadenitis, within the last 2,600 to 28,000 years (6, 7, 10–12). The apparent question is how did *Y. pseudotuberculosis* evolve into such a virulent, dangerous and remarkably different pathogen, *Y. pestis*? Could there still be an ancestral *Y. pseudotuberculosis* circulating in China that might hold clues to the evolution to *Y. pestis?*

**Figure 1:**
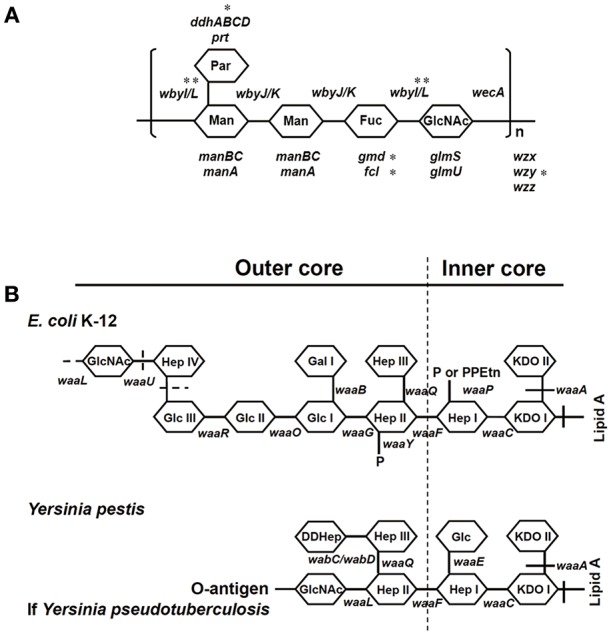
Structures of O-antigen of *Y. pseudotuberculosis* and core LPS. **(A)** Genes involved in the biosynthesis of O-antigen of *Y. pseudotuberculosis*. ^*^The genes were sequenced in this study. **(B)** Genes involved in the biosynthesis of core LPS are shown at their approximate site of action (solid line). The sites, which are variably substituted or still under investigation, are indicated by dashed lines. Fuc, L-fucose; Gal, galactose; GalNAc, N-Acetyl-galactosamine; Glc, glucose; GlcNAc, N-Acetylglucosamine; Hep, heptose; KDO, 2-keto-3-deoxyoctonate; Man, mannose; P, phosphate; Par, Paratose; PEA, phosphoethanolamine; PPEtn, phosphoethanolamine. It should be noted that *E. coli* K12 and *Y. pestis* naturally do not possess an O-antigen.

A distinguishing difference between these pathogens is that *Y. pseudotuberculosis* contains an O-antigen of lipopolysaccharide (LPS), which was lost by *Y. pestis* during its evolution (13–15) (Figure 1B). LPS plays a major role in the pathogenicity of Gram-negative bacterial pathogens including pathogenic species of the genera *Escherichia, Shigella, Klebsiella, Yersinia*, and *Salmonella*. The presence of LPS promotes toxicity as well as resistance to phagocytosis and serum-dependent (complement-dependent) killing (16–21). LPS generally consists of three structural regions: (i) the lipid A backbone, (ii) an oligosaccharide core (core LPS), and (iii) the somatic O-polysaccharide outer region (also called O-antigen, O-specific antigen, or O-specific side chain) (Figure 1A). Gram-negative bacteria are classified as smooth or rough based on the presence or lack of the O-antigen (O-Ag), respectively. *Y. pestis* does not contain an O-antigen (14, 15) and therefore the shortened LPS is also referred to as lipooligosaccharide (LOS). Why would *Y. pseudotuberculosis* sacrifice the production of O-Ag, one of its key virulence factors, during the evolution to *Y. pestis*?

We have shown that human DC-specific intercellular adhesion molecule-grabbing nonintegrin (hDC-SIGN, CD209a), a C-type lectin receptor on antigen presenting cells (APCs) such as macrophages and dendritic cells (DCs), is a receptor for the core LPS of several Gram-negative bacteria, including *E. coli, Haemophilus ducreyi, Neisseria gonorrhoeae, Yersinia* spp., and *Salmonella typhimurium*, promoting bacterial adherence and phagocytosis (22–27). In addition, *Y. pseudotuberculosis*, via its core LPS-CD209 interaction, may hijack APCs to be disseminated to lymph nodes, spleen and liver (28).

Moreover, hDC-SIGN (CD209a) is a receptor for HIV gp120 that uses DC-SIGN to be captured and trafficked to target cells such as CD4^+^ T cells (29–31). Mouse DC-SIGN-related protein 1 (SIGNR1, CD209b), expressed on splenic marginal zone, lymph nodes, and peritoneal macrophages, plays a role in lymphocyte migration from the blood into tissues. Here, we show that SIGNR1 is a cellular receptor for *Y. pestis* and that an exposed core LPS is essential for the APCs/*Y. pestis* interaction, host dissemination and infection. Therefore, it is possible that the loss of expression of O-antigen during evolution from *Y. pseudotuberculosis* might have endowed *Y. pestis* the ability to hijack APCs in rodents in order to spread into lymph nodes and initiate host infections (32, 33).

## Results

### From the Perspective of O-Antigen Synthesis Genes, *Y. pestis* Might Have Evolved From One of Several Specific Evolutionary Branches of *Y. pseudotuberculosis*

**Figure 2:**
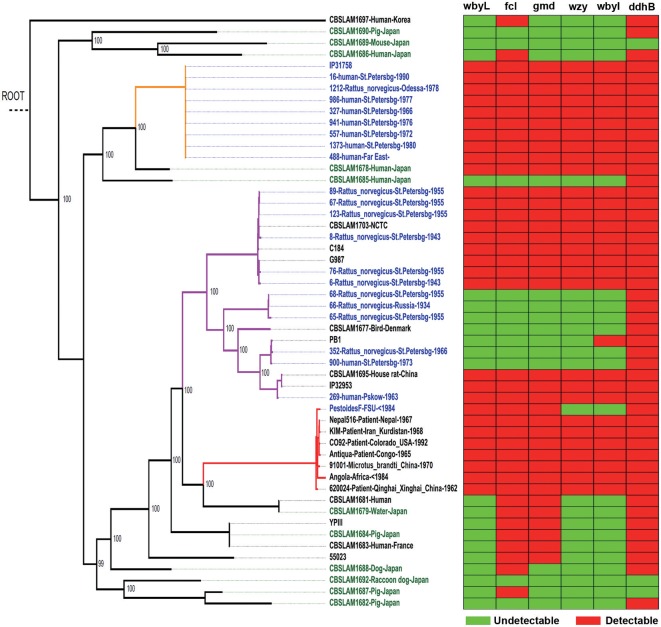
*Y. pseudotuberculosis* rather than *Y. pestis*, isolated from China, show the multiple mutations of O-antigen synthesis genes. In the heatmap, rows represent six O-antigen synthesis genes, and columns represent data from *Y. pseudotuberculosis* and *Y. pestis* strains. Red cell indicates the gene was detectable in this strain while green cell indicates the gene was undetectable. In the phylogenetic tree, red branches are *Y. pestis*, and the rest are *Y. pseudotuberculosis*.

*Y. pseudotuberculosis* strains possess an O-antigen, the production of which was lost by *Y. pestis* during evolution. Based on a recent study on the evolution of *Y. pseudotuberculosis* (12), we sequenced six O-antigen synthesis genes (Figure 1A) just outside of *waaL* (Figure 1B) of 39 strains of *Y. pseudotuberculosis* and eight strains of *Y. pestis* to investigate the changes in these genes during evolution (12). As shown in a summary (Figure 2), unlike *Y. pestis*, the genes from the *Y. pseudotuberculosis* strains showed extreme diversity, which echoes the conclusion of the Cui's study (12). However, there are several new findings; (1) *wbyL*: Only one *Y. pestis* strain from the eight analyzed showed a non-synonymous mutation in *wbyL* compared to *Y. pseudotuberculosis*. (2) *wbyI*: The *wbyI* genes from all eight *Y. pestis* strains appeared to have lost their potential functions due to the loss of a 62 base pair fragment presents in *Y. pseudotuberculosis*. (3) *gmd*: The *gmd* gene in all eight *Y. pestis* strains appeared to be non-functional due to an insertion. (4) *fcl*: Only one strain of the eight *Y. pestis* strains (Orientalis, the cause of third pandemic of plague) analyzed showed a deletion. The other seven carried a fully functional gene. (5) *wzy*: Except for one strain (Pestoides F), all the strains analyzed appeared to have lost the function of this gene. (6) *ddhB*: Except for Pestoides A (0.PE4c) and Microtus (0.PE4i), all lost function due to a frame-shift mutation. It is reported that lack of O-antigen is essential for plasminogen activation and invasiveness of *Y. pestis* (34). Therefore, loss of genes involved in O-antigen synthesis in *Y. pestis* affects its function.

In short, based on the detailed analysis of O-antigen synthesis genes, we showed again that *Y. pestis* might evolve from one of several specific evolutionary branches of *Y. pseudotuberculosis*.

### The Core LPS of *Y. pestis* Is Involved in the Invasion of Mouse Macrophages

**Figure 3:**
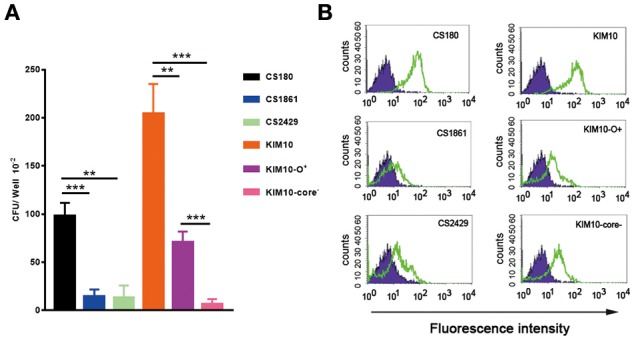
Interaction of *Y. pestis* with mouse peritoneal macrophages involves its core LPS. **(A)** Gentamicin protection- and **(B)** flow cytometry-based assays were used to determine the invasion rate of these sets of *E. coli* K-12(CS180, CS1861, and CS2429) and *Y. pestis* KIM10 (Δ*ail*, Δ*ail*-O^+^ and Δ*ail*-Core^−^) into purified mouse peritoneal macrophages. **(B)** CFDA-SE labeled and unlabelled bacteria are indicated by open and filled symbols, respectively. Data are representative of three independent experiments.

Although the O-antigen synthesis genes in *Y. pseudotuberculosis* are diverse, the core LPS appears to be identical to that of *Y. pestis* (Figure 1B) (35, 36). Several Gram-negative bacterial strains use their core LPS to interact with human DCs (22–24, 28). Furthermore, another study showed that N-acetylglucosamine (GlcNAc) within the core LPS (Figure 1B) may mediate the interaction with DCs (24). To investigate the hypothesis that *Y. pestis* might also use its core LPS to interact with mouse macrophages, *Y. pestis* KIM10-Δ*ail* (a natural rough strain with the core LPS exposed), and its two isogenic derivatives; KIM10-Δ*ail*-O^+^ (a smooth strain in which the outer-core LPS is shielded by O-antigen) and KIM10-Δ*ail*-Core^−^ (with truncated LPS outer-core, i.e., a deep rough strain) were examined for their ability to invade mouse macrophages. Three corresponding *E. coli* K-12 strains: CS180 (rough), CS1861 (CS180 expressing an O-antigen, smooth), and CS2429 (deep rough) were used as controls. We have used this set of strains in previous studies that demonstrate that the exposure of the core LPS by several Gram-negative bacteria is essential to initiate contact with human DCs (22–24). Figure 3A shows that *Y. pestis* KIM10-Δ*ail* (rough) and *E. coli* K12 180 (rough) invade mouse macrophages. In contrast, both deep rough and smooth strains resulted in a reduced level of invasion of mouse macrophages in both assays (Figures 3A,B). All strains used were cultured at 26°C, at which *Y. pestis* does not produce the F1 capsule that blocks interaction with host cells (37, 38). This result indicates that phagocytosis of these bacteria by mouse macrophages involves the core LPS ligand. The fact that the O-antigen-expressing and the deep rough-*Y. pestis* still interact with these host cells, although at a reduced level, suggests that besides the core LPS, other components of *Y. pestis* also mediate interactions with macrophages.

In addition, deep rough mutants of Gram-negative bacteria in general are more sensitive to biological killing (39, 40). This fact should be taken into consideration in interpreting the data from the gentamicin survival assay of KIM10-Δ*ail*-Core^−^ and CS2429 (Figure 3A).

### Murine SIGNR1 Is a Receptor for the Core LPS of *Y. pestis*

#### Signr1 (CD209b), but Not the mDC-SIGN (CD209a) Is a Receptor for the Core LPS of *Y. pestis*

**Figure 4:**
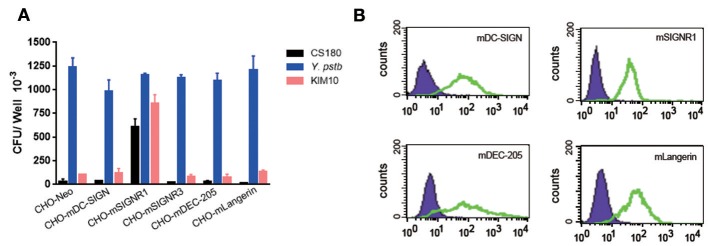
*Y. pestis* invades CHO-SIGNR1, but not other CHO transfectants. **(A)** The invasion of three bacteria; *E. coli* K-12 CS180, *Y. pseudotuberculosis* (Y1) and *Y. pestis* KIM10-Δ*ail* into several CHO transfectants with the expression of mouse C-type lectins; CHO-mDC-SIGN, CHO-SIGNR1, CHO-SIGNR3, CHO-mDEC-205 (CD205), and CHO-mLangerin (CD207). CHO transfectants were incubated with the bacterial strains for 2.5 h and the extracellular bacteria were killed by 100 μg/ml of gentamicin. The number of intracellular bacteria was determined by counting the recovered CFUs. **(B)** The expression of level of each transfectant and CHO-Neo was shown in open and filled curve, respectively. Data are representative of three independent experiments.

To determine if the invasion of *Y. pestis* into mouse macrophages was a result of the interaction between *Y. pestis* and murine DC-SIGN (mDC-SIGN), five CHO transfectants stably expressing the mouse C-type lectin receptors mDC-SIGN, SIGNR1, SIGNR3, mDEC-205 (CD205), and mLangerin (CD207) (41, 42) (Figure 4A) were infected with *Y. pestis* KIM10-Δ*ail* and CS180. *Y. pseudotuberculosis* (Y1) grown at 26°C was used as a positive control in this experiment because it invades most epithelial cell lines, including CHO (43), via the invasin-integrin interaction (44, 45). The expression of each C-type lectin is shown in Figure 4B. CHO-SIGNR1, but not other C-type lectin transfectants including CHO-mDC-SIGN, efficiently phagocytized *Y. pestis* KIM10-Δ*ail* and CS180 (Figure 4A). Since macrophages from the mouse peritoneal cavity express SIGNR1 (Figure 8B) (41, 42), it is anticipated that the phagocytosis of *Y. pestis* by these macrophages involves the SIGNR1-core LPS interaction.

#### GlcNAc Epitope Within the Core LPS of *Y. pestis* May Play a Role in Interaction With SIGNR1

**Figure 5:**
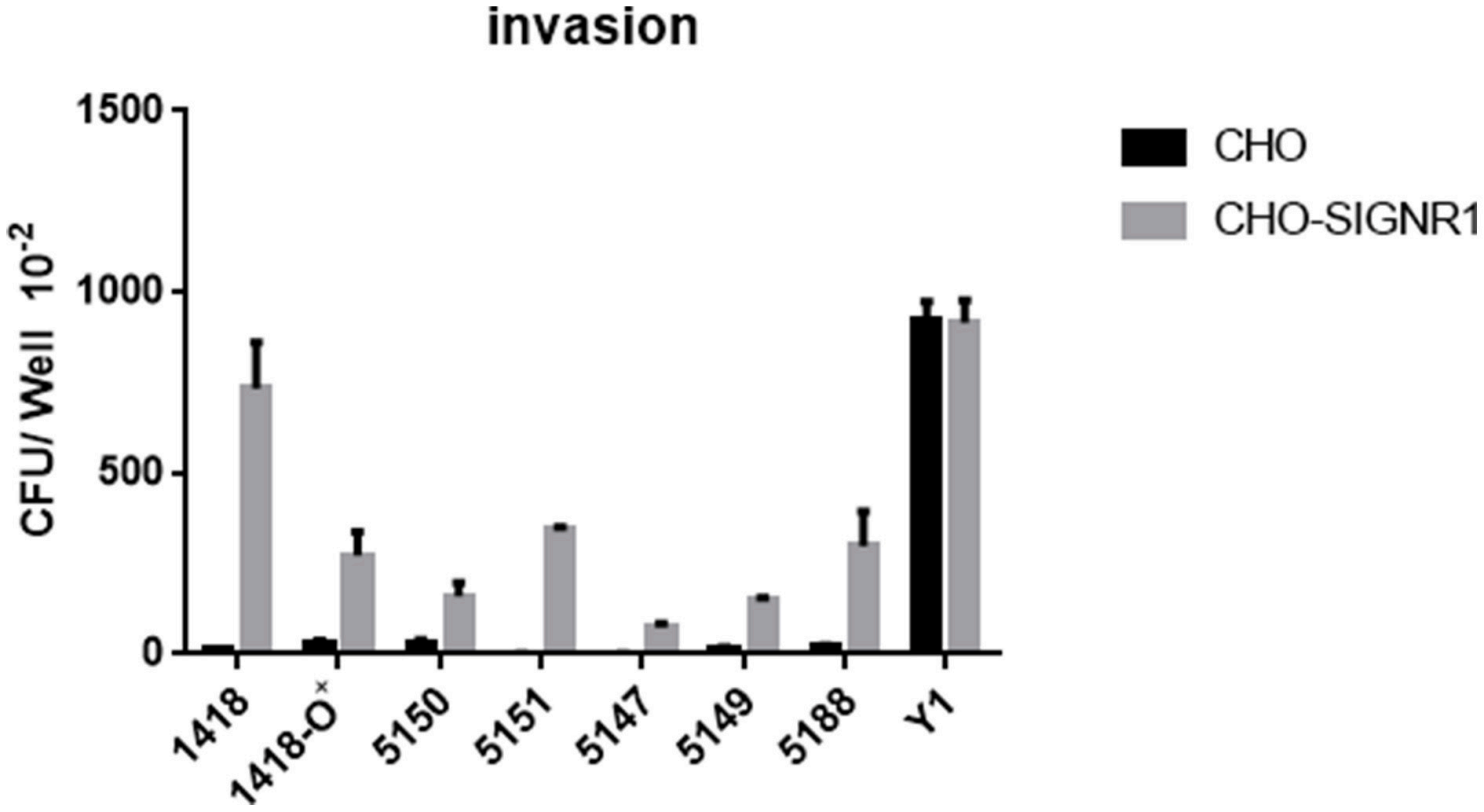
A specific sugar epitope within core LPS may be responsible for interacting with SIGNR1. *Y. pestis* 1418 (D27, wild type) and its mutants (1418-O+, 5147 D27-Δ-*waaQ*, 5149 D27-Δ-*waaE*, 5150 D27-Δ-*waaL*, 5151 D27-Δ-*wabD*, and 5188 D27-Δ-*waaA*) that possess specific sugar epitope within core LPS were used to determine the invasion rate. *Y. pseudotuberculosis* (Y1) were used as control strains. Data are representative of three independent experiments.

To confirm that a specific sugar epitope within the core LPS is responsible for interacting with SIGNR1, another set of core LPS mutants from *Y. pestis* strain D27 (46) was utilized (Figure 1B and Table 1). Consistent with the results obtained for *E. coli* (22), *Salmonella* (24)*, Neisseria gonorrhoeae* (23)*, Neisseria meningitidis* (52), *H. ducreyi* (24), and *Y. pseudotuberculosis* (28), the rough *Y. pestis* strain (wild-type) (25), rather than the O-antigen expressing *Y. pestis* promoted a typical SIGNR1-mediated adherence and phagocytosis (Figure 5). Furthermore, the deletion of the GlcNAc epitope in the *waaL* mutant (Figure 1B) reduced the ability of *Y. pestis* to interact with CHO-SIGNR1, indicating again that the core LPS is the ligand. Interestingly, phagocytosis of the *Y. pestis* mutants by CHO-SIGNR1 was consistent with the idea that the lengthier the core LPS is, the greater the ability to promote phagocytosis, as shown with *Salmonella* (24).

**Table 1:** Bacterial strains, cell lines used in this study.

**Strains**	**Genotypes (phenotypes)**	**References**
***Yersinia pestis***		
KIM6	Lcr–, Pgm+, Pst+, wild type (rough)	(47)
KIM10-Δ*ail*	Derivative of KIM5 in which the *ail* gene has been deleted and both plasmid pCD1 and pPCP1 have been cured	(48)
KIM10-Δ*ail*-O+	KIM10-Δ*ail* expessing O-antigen	This work
KIM10-Δ*ail*-Core-	Deep rough mutant derivative of KIM10-Δ*ail*, Truncated LPS outer-core	This work
*Y. pestis* 1418	KIM D27 (*Lcr^+^, Pgm−, Pst+*) transformed with pBR322 plasmid	This work
*Y. pestis* 1418-O*^+^*	*Y. pestis* 1418 transformed with pAY100.1 plasmid expressing an O-antigen from *Y. enterocolitica* serotype O:3	This work
*Y. pestis* 5150	KIM D27-Δ*waaL*	(46)
*Y. pestis* 5151	KIM D27-Δ*wabD*	(46)
*Y. pestis* 5147	KIM D27-Δ*waaQ*	(46)
*Y. pestis* 5149	KIM D27-Δ*waaE*	(46)
*Y. pestis* 5188	KIM D27-ΔwaaA	(46)
*Y. pestis* 91001	A fully virulent to mice	(49)
***Y. pseudotuberculosis***		
Y1	Serotype O:1a, wild-type expressing invasin, but with pYV plasmid naturally cured (smooth)	(43)
***E. coli K-12***		
CS180	Contains core LPS but lacks O-antigen (rough)	(50, 51)
CS1861	CS180 expressing O-antigen (smooth)	(50, 51)
CS2429	Lacking both O-antigen and most of core (*waaC*)	(50, 51)
**Cell lines**	**Characteristics**	
HeLa-NEO cells	Control cell line, which expresses the neomycin resistance gene only	
HeLa-hDC-SIGN	Generated by transfecting HeLa cells with human DC-SIGN cDNA for stable surface expression	
CHO-NEO cells	Control cell line, which expresses the neomycin resistance gene only	
CHO-SIGNR1 cells	Generated by transfecting CHO cells with mouse SIGNR1 cDNA for stable surface expression	
CHO-mDC-SIGN cells	Generated by transfecting CHO cells with mouse DC-SIGN cDNA for stable surface expression	
CHO-SIGNR3 cells	Generated by transfecting CHO cells with mouse SIGN-R3 cDNA for stable surface expression	
CHO-CD205 cells	Generated by transfecting CHO cells with mouse CD205 cDNA for stable surface expression	
CHO-CD207 cells	Generated by transfecting CHO cells with mouse CD207 cDNA for stable surface expression	
Primary macrophage	Primary macrophages from mouse peritoneal cavity	
CRL-2455	Alveolar macrophage cell line	
J774A.1	Macrophage cell line expressing SIGNR1	

The results from **Figures 3–5** lead to three conclusions. First, SIGNR1 is a receptor for *Y. pestis*. Second, the core LPS of *Y. pestis* as a ligand is involved in this interaction. Third, GlcNAc is important for the SIGNR1 interaction (24). In fact, the data published 2015 in Immunology and Cell Biology (27), regarding the interaction of human CD207, awarded an editorial comments, entitled “A new cellular target for *Yersinia pestis*” (53).

### *Y. pestis* Invades Mouse Macrophage Cell Lines That Express Limited SIGNR1

**Figure 6:**
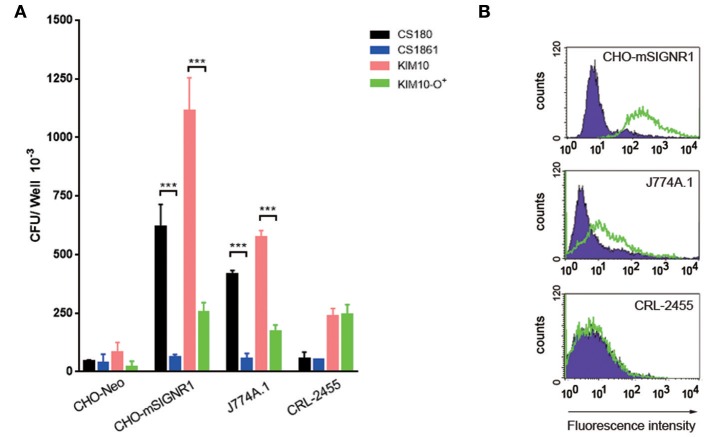
SIGNR1-mediated phagocytosis depends in part on core LPS. **(A)** The two sets of bacteria; *E. coli* K-12 strain (CS180 and CS1861) and *Y. pestis* KIM10-Δ*ail* (Δ*ail* and Δ*ail*-O^+^) were tested for whether the SIGNR1-mediated interaction is depended on this core LPS with CHO transfectants and macrophage cell lines J774A.1 and CRL-2455. **(B)** The expression of SIGNR1 of these macrophage cell lines and control CHO-SIGNR1. Data are representative of three independent experiments. ^***^P < 0.001.

*Y. pestis* invades certain macrophage cell lines, such as the J774A.1 (54, 55). In order to determine if the same ligands are involved in this interaction as was found with primary macrophages (Figure 3A) and CHO-SIGNR1 (Figures 4, 5), the KIM10-Δ*ail* and KIM10-Δ*ail*-O^+^ were examined for their ability to invade two macrophage cell lines, J774A.1, and CRL-2455 (Figure 6A). The expression level of SIGNR1 on J774A.1 is shown in Figure 6B, but the expression of this receptor on CRL-2455 cell line was undetectable (Figure 6B).

The interaction of the KIM10-Δ*ail* and KIM10-Δ*ail*-O^+^ with J774A.1 resembled the results obtained using primary macrophages. KIM10-Δ*ail* invades host cells better than KIM10-Δ*ail*-O^+^, and CS180 is also able to invade J774A.1 cells. However, the CRL-2455 cell line, which does not express SIGNR1, is still able to phagocytose low levels of both KIM10-Δ*ail* and KIM10-Δ*ail*-O^+^, but not CS180, indicating that the core LPS does not interact with the CRL-2455 cell line.

In short, these results indicated that other components or mechanisms can also lead to internalization of *Y. pestis* by macrophages, besides the core LPS-SIGNR1 interaction. Because CS180 invades SIGNR1-expressing macrophage cell line, but not the CRL-2455, it also confirms the core LPS-SIGNR1 interaction with macrophages.

### The Inhibition of SIGNR1-Mediated Phagocytosis of *Y. pestis* by Anti-SIGNR1 Antibody, Mannan, Peptides, and Oligosaccharides

**Figure 7:**
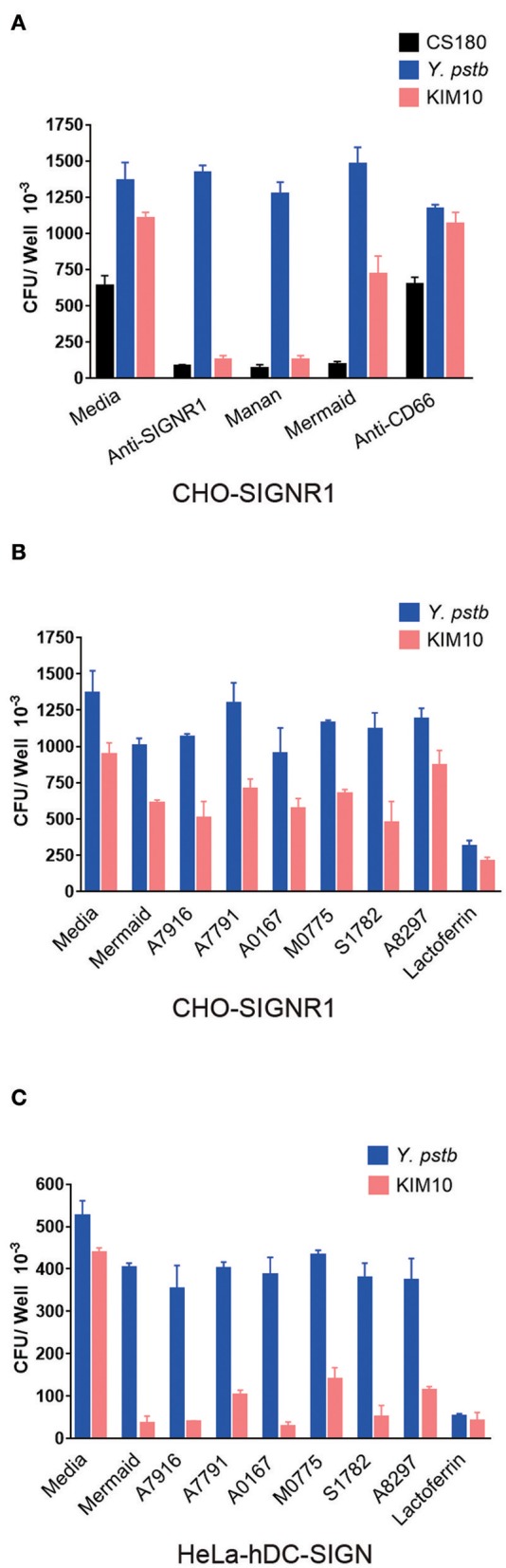
The inhibition of SIGNR1-mediated phagocytosis of *Y. pestis* by anti-SIGNR1 antibody, mannan, peptides, and oligosaccharides. **(A)** *Y. pestis* KIM10-Δ*ail* were incubated with CHO-SIGNR1 in the presence or absence of anti-CD66, anti-SIGNR1, mannan and DC-SIGN-like protein (His-Mermaid). The phagocytosis rate of *Y. pestis* was evaluated by the recovery of bacteria from gentamicin protection. *E. coli* K12 180 and *Y. pseudotuberculosis* serotype O:1b were used as control strains to show core LPS-dependent or independent interaction with CHO-SIGNR1. **(B,C)** Various oligosaccharides and lactoferrin were also tested for their ability to inhibit the interaction of CHO-SIGNR1 and HeLa-hDC-SIGN with *Y. pestis* KIM10-Δ*ail* and *Y. pseudotuberculosis* sero-type O:1b (*Y. pstb*). Data are representative of three independent experiments.

To verify the specificity of the interaction of *Y. pestis* with SIGNR1, we examined whether the core LPS-SIGNR1 interaction could be inhibited by SIGNR1 antibody, mannan, His-Mermaid and CD66 antibody. Mannan is a well-documented reagent for its ability to block the DC-SIGN-mediated interactions with HIV. His-Mermaid is the recombinant form of Mermaid, a newly identified DC-SIGN-like protein (56) that has previously been shown to inhibit the core-LPS-hDC-SIGN interaction (24). Anti-CD66 antibody was employed as a control antibody. *E. coli* K12 CS180 and *Y. pseudotuberculosis* serotype O:1b, mediating a SIGNR1-dependent and -independent interaction, respectively, were again utilized as control strains. Figure 7A shows that the anti-SIGNR1 and mannan inhibit the interaction between *Y. pestis* or CS180 and CHO-SIGNR1, indicating a specific interaction between SIGNR1-*Y. pestis*, which promotes the invasion of this bacteria into mouse APCs.

Mermaid possesses the ability to inhibit hDC-SIGN-mediated interaction with several Gram-negative bacteria (24), but the inhibition of the *Y. pestis*-SIGNR1 interaction by Mermaid is limited (Figure 7A), suggesting that hDC-SIGN and SIGNR1 have distinguishing features employed during interactions with core LPS of *Y. pestis* (41, 42).

Certain oligosaccharides inhibit CS180-HeLa-hDC-SIGN-promoted interaction (24). Figures 7B,C show that although these reagents have very limited abilities to inhibit the SIGNR1-mediated interaction, but the β-D-Gal-(1 → 3)-D-GalNAc (A0167), β-D-Gal-(1 → 6)-D-GlcNAc (A7916) and α-NeuNAc-(2 → 3)-β-D-Gal-(1 → 4)(α-L-Fuc-)-D-GlcNAc (S1782) oligosaccharides inhibit the core-LPS-hDC-SIGN interaction of *Y. pestis* KIM10-Δ*ail* very well (25). The recovery rates of Y1 and KIM10-Δ*ail* bacteria in both cell lines are dramatically reduced in the presence of lactoferrin, indicating that these bacteria are killed by this peptide, which is well-known for its ability to kill bacteria (57).

In short, the results also indicated that hDC-SIGN (hCD209a) and SIGNR1 (CD209b) are different in terms of their interactions with *Y. pestis*.

### *In vivo* Phagocytosis of *Y. pestis* by Mouse Macrophages Involves the Core LPS

*Yesinia pestis* can invade macrophages as well as other APCs during infection in mice (32, 58, 59). To test whether the core LPS-mediated interaction also occurs *in vivo*, we injected bacterial suspensions directly into the mouse peritoneal cavity. This approach is analogous to our previous study showing that the interaction of mouse CD205 (DEC-205) receptor on alveolar macrophages with the *Y. pestis* plasminogen activator (Pla) occurs *in vivo* (26).

**Figure 8:**
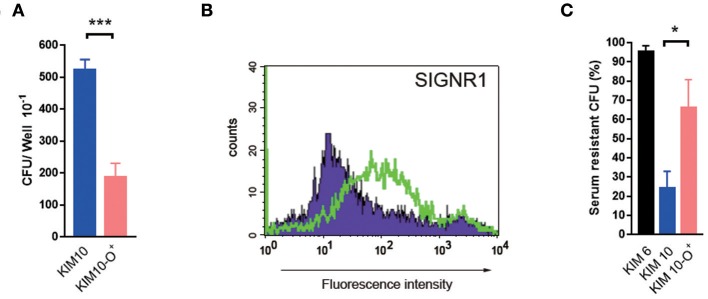
Core LPS-SIGNR1 interaction with macrophages occurs *in vivo*. *Y. pestis* (KIM10-Δ*ail* and KIM10-Δ*ail*-O^+^) were tested for their ability to invade macrophages *in vivo*. **(A)** Bacterial suspensions were inoculated into the mouse peritoneal cavity. After incubation for 1.5 h, macrophages were examined for the rate of internalized bacteria. **(B)** The SIGNR1 expression of the mouse peritoneal macrophages. **(C)** KIM10-Δ*ail*-O+ was more resistant than KIM10-Δ*ail* in serum resistant assay. ^*^P < 0.05, ^***^P < 0.001. Data are representative of three independent experiments.

After 1.5 h of infection, the intraperitoneal fluids or exudates were collected and placed in gentamicin media to kill the extracellular bacteria. Figure 8A shows that a higher percentage of viable *Y. pestis* was recovered compared to the O-antigen expressing-*Y. pestis*, KIM10-Δ*ail*-O^+^. This increased recovery rate of KIM10-Δ*ail* is not due to the ability of the O-antigen to protect against complement-mediated killing (16–21), as in the serum killing assay KIM10-Δ*ail*-O^+^ was more resistant than KIM10-Δ*ail* (Figure 8C). In short, because KIM10-Δ*ail*-O^+^ was more resistant than KIM10-Δ*ail*, the increased recovery of KIM10-Δ*ail* suggests that core LPS-mediated phagocytosis of *Y. pestis* occurs *in vivo*.

### O-Antigen Expressing *Y. pestis* Have a Reduced Ability to Be Disseminated to Lymph Nodes

Our recently published data showed that *Y. pseudotuberculosis* uses its core LPS to interact with hDC-SIGN and SIGNR1 receptors, leading to its dissemination (28). We therefore hypothesized that the dissemination of *Y. pestis* to the lymph node (LN), spleen and liver would also be facilitated by this host-pathogen interaction. Subsequently, if the exposed core LPS of *Y. pestis* could be shielded that should reduce dissemination.

#### Higher Numbers of Rough Rather Than Smooth *Y. pestis* Bacteria Were Disseminated to LN, Spleen, and Liver

**Figure 9:**
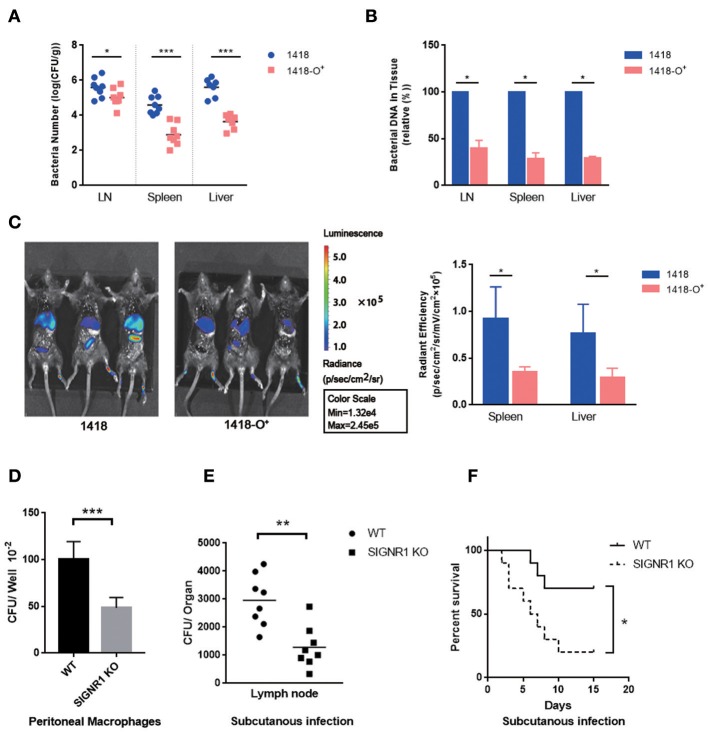
O-antigen expressing *Y. pestis* have a reduced ability to be disseminated to lymph nodes. **(A)** *Y. pestis* 1418 and 1418-O^+^ were examined for their ability to be disseminated to lymph node, spleen and liver. **(B)** Bacterial load determined by real time PCR. **(C)** Dissemination of *Y. pestis* 1418 and 1418-O^+^ with pXEN-18 were monitored by the bioluminescence at 72 h post bacterial inoculation into hind paws of mice. **(D)** Reduction the phagocytosis of *Y. pestis* 1418 by peritoneal macrophages of SIGNR1 KO mice. **(E)** Reduction in *Y. pestis* 1418 dissemination to lymph node in *Y. pestis* infected SIGNR1 KO mice at 5 hpi as compared to infected WT mice. **(F)** Survival rates of WT mice and SIGNR1 KO mice after the infection of *Y. pestis* 1418. For each group, 10 mice were infected with 10^8^ CFU *Y. pestis* 1418 and observed until 15 days post infection. Log-rank test was performed. The data shown are obtained from the three independent experiments. ^*^P < 0.05, ^**^P < 0.01, ^***^P < 0.001.

To achieve a *Y. pestis* strain producing smooth LPS *in vivo*, we introduced plasmid pAY100.1 into strain *Y. pestis* 1418. Plasmid pAY100.1 carries the O-Ag gene cluster of *Y. enterocolitica* serotype O:3 and produces the O-Ag (25, 27, 60, 61). *Y. pestis* 1418 (KIM D27) is a conditionally virulent strain, which is able to cause typical plague in mice depending on the route of infection and dose (Figure 9A) (46). As a control, the plasmid vector pBR322 was transformed to *Y. pestis* 1418, which was described in the previous publications (25–27). Mice were infected via injection into hind paws and sacrificed after 72 h. LN, spleen and liver were then homogenized. The dissemination rates of the bacteria into the different organs were calculated by plating and counting CFUs and fluorescent intensity. Figures 9A,C show that higher numbers of 1418 than 1418-O^+^ bacteria were isolated from LN, spleen, and liver. It should be recognized that both strains exhibited no differences in growth and adhered to both HeLa and CHO cells (data not shown).

#### Higher Quantity of Bacterial DNA Was Detected in MLNs, Livers and Spleens of Mice Infected With Rough Than Smooth *Y. pestis*

The mice were challenged as described above, but the mice were sacrificed after 8 h infection. The *ail* gene DNA of *Y. pestis* in LN, spleen and liver was quantitated by real time PCR. The bacterial load was higher in mice infected with *Y. pestis* 1418 than in *Y. pestis* 1418-O^+^ (Figure 9B), which was consistent with the data of bacterial recovery assay described above.

#### Evaluation of Dissemination With Bioluminescence Imaging

C57BL/6J mice were subcutaneously inoculated in hind paws with *Y. pestis* 1418 or 1418-O^+^ transformed with the pXEN-luxCDABE (pXEN-18) plasmid, and bioluminescent signals were monitored at 0, 48, and 72 h post inoculation (hpi). The bioluminescent scale ranges from most intense (red) to least intensity (violet) (Figure 9C). All images are standardized to the same radiance scale. Bioluminescence was detected in the abdomen and thoracic region of the mice at 48 hpi (data not shown), but the highest level of signal was observed in the region corresponding to liver and spleen at 72 hpi. Signals from the mice infected with *Y. pestis* 1418-O^+^ were significantly increased in intensity than those in *Y. pestis*1418 group, suggesting the dissemination ability of O-antigen expressing *Y. pestis* was reduced.

#### The Phagocytosis of *Y. pestis* by Macrophages and Host Dissemination in SIGNR1 Knock-Out Mice Were Reduced

To demonstrate the involvement of SIGNR1 in the interaction of *Y. pestis* and macrophages *in vivo*, we evaluated the phagocytic and intracellular killing capacity of peritoneal macrophages derived from SIGNR1 knock-out (SIGNR1) mice. The phagocytosis of *Y. pestis* by peritoneal macrophages (Figure 9D) and host dissemination (Figure 9E) in by SIGNR1 KO mice were significantly reduced, indicating the direct involvement of SIGNR1. However, the SIGNR1 KO mice were shown more susceptible to infection of *Y. pestis* 1418 (Figure 9F), which is addressed in Discussion.

In summary, the results suggest that *Y. pestis* could utilize its core LPS to interact with SIGNR1 to enhance the dissemination in host tissues.

### The Expression of O-Antigen Reduces the Infectivity of *Y. pestis*

**Figure 10:**
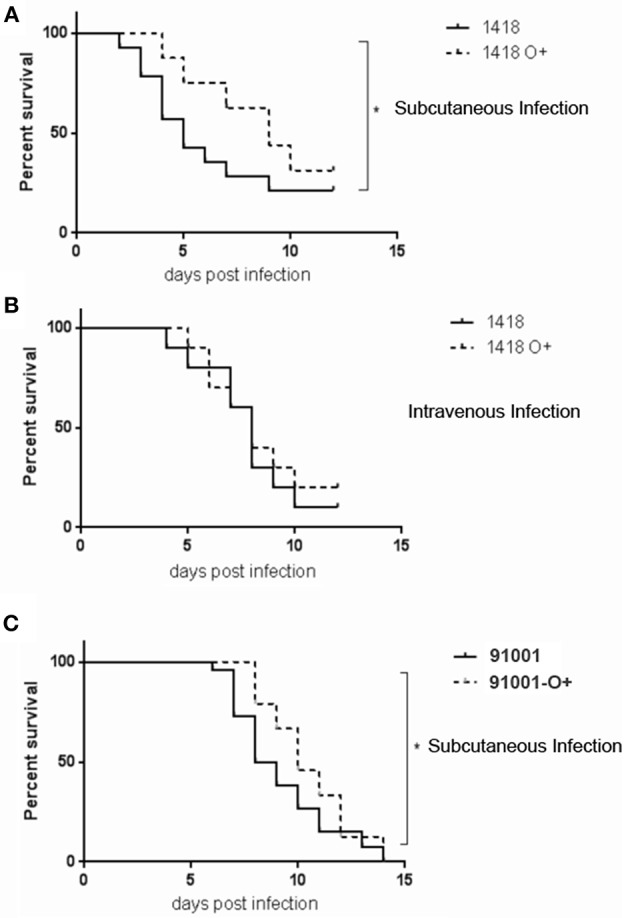
The expression of O-antigen reduces death rates by infection. **(A,B)** Mice were subcutaneously and intravenously inoculated with *Y. pestis* 1418/1418-O^+^. **(C)** Mice were subcutaneously inoculated with *Y. pestis* 91001. The rates of mortality were recorded every 12 hpi. ^*^P < 0.05.

To examine whether the reduced dissemination, when the interaction of core LPS-SIGNR1 interaction was blocked by expression of O-antigen, leads to reduction of infection, C57BL/6J mice were challenged with two sets of *Y. pestis* 1418 and 91001. Survival analyses following subcutaneous injection, the route bubonic plague, revealed that the mice infected with *Y. pestis* 1418 suffered from a significant survival disadvantage compared to those infected with *Y. pestis* 1418-O^+^ (Figure 10A). However, there is no difference with the intravenous inoculation which mimic the septicemic plague (Figure 10B). *Y. pestis* 91001, a fully virulent strain isolated from China (62), was used to challenge each mouse with 30 CFU via subcutaneous inoculation. When cover the core LPS of *Y. pestis* 91001 with the expression of O-antigen, the mice infected with *Y. pestis* 91001-O^+^ displayed significant survival advantage relative to *Y. pestis* 91001 (Figure 10C). The results shown above demonstrate that the exposure of core LPS is important for *Y. pestis* in host dissemination and bacterial infection.

## Discussion

*Y. pestis*, the cause of plagues, has directly evolved from *Y. pseudotuberculosis* within the last 2,600 to 28,000 years (7, 10, 11). It is well-documented that a distinguishing characteristic between these two *Yersinia* species is that *Y. pseudotuberculosis* strains possess an O-antigen (intact LPS expression), which was lost by *Y. pestis* during evolution. As a result, after entering the skin by an infected flea, *Y. pestis*, with its core LPS, can directly interact with APCs, leading to phagocytosis of the pathogen (25). The infected APCs consequently serve as a Trojan Horse to deliver the *Y. pestis* to lymph nodes (33) and initiate the plagues. In this study, we demonstrated that it is the murine SIGNR1 that serves as a receptor for the core LPS of *Y. pestis* to promote bacterial dissemination and infection.

The current model for the initial stages in the pathogenic process mediated by *Y. pestis* is reminiscent of how HIV-1 infects hosts. It is well-established that HIV-1 pirates DC-SIGN (CD209), a C-type lectin receptor expressed by APCs, to be captured and transmitted to target cells such as CD4^+^ lymphocytes (29–31).

The connection between *Y. pestis* and HIV extends to another receptor, CCR5, which is a co-receptor for HIV. There is evidence that a certain subpopulation of Caucasians is resistant to HIV infection because of their natural deletion of this receptor. It has been suggested that human populations have also been selected by *Y. pestis*, based on its sensitivity to human CCR5 (63). Therefore, CCR5 knock-out mice were challenged with *Y. pestis*, but no protection was observed (64, 65).

Besides showing that *Y. pestis* uses its core LPS to interact with SIGNR1 *in vitro*, we designed and performed an *in vivo* interaction assay to determine whether the core LPS-SIGNR1 interaction occurs *in vivo* between *Y. pestis* and macrophages. Even so, the fundamental question remains as to whether core LPS-SIGNR1 interaction plays a role in *in vivo* infections.

The straightforward approach is to test if SIGNR1 knock-out mice would be resistant to plague, as KO mice have been used successfully to identify viral receptors. For example, the CEACAM1 (CD66a) receptor KO mice have an increased resistance to mouse hepatitis viral infection (66), because mouse CEACAM1 is a receptor for mouse hepatitis virus (67). However, there are potential limitations of this approach to study bacterial-host cell interactions. Strangely, there are no credible receptor knock-out models that are more resistant to bacterial infection. The reason might be simple; viral infection is less complicated than bacterial infections. One receptor might not be enough to determine the fate of a bacterial infection, which might contribute to the failure of CCR5-knock-out mice to resist *Y. pestis* infection (64, 65), even if CCR5 were a receptor for *Y. pestis*. In addition, SIGNR1 knock-out mice are more susceptible to bacterial infection (68), probably because of the role of this receptor in the complement pathway (69). In short, this strategy could only work if SIGNR1 is the only, or a very prominent, receptor for *Y. pestis*. Unfortunately, many pathogens do not depend on only one receptor in their interactions with host cells.

The oligosaccharides and small peptide were chosen to analyse their ability to inhibit *Y. pestis*-C-type lectin interactions for the four following rationales: (1) Oligosaccharides that interfere with the interaction between host cells and *Y. pestis* have been examined for their therapeutic potential (70, 71). (2) Several Gram-negative bacteria might use their core LPS, consisting of oligosaccharides, to interact with hDC-SIGN, which could be inhibited by oligosaccharides (24). (3) SIGNR1 binds to the capsular polysaccharide of *Streptococcus pneumoniae* (68, 69, 72). (4) HIV uses gp 120-DC-SIGN interaction to be captured by DCs and transmitted to CD4^+^ cells (29–31). Therefore, blockage of DC-SIGN-mediated transmission of HIV has been undertaken by many investigators in order to find therapeutic strategies for HIV infection. For example, lactoferrin, a small peptide from milk, and Lewis X components (oligosaccharides) have been shown to prevent DC-mediated HIV-1 transmission by blocking the DC-SIGN-gp120 interaction (73, 74). Interestingly, our data show that some oligosaccharides indeed inhibit the interaction between *Y. pestis* and hDC-SIGN.

Taken together, this study has demonstrated that SIGNR1 is a cellular receptor for *Y. pestis* and possibly plays a role in host dissemination and bacterial infection. Since hDC-SIGN and SIGNR1 share a similar ability to interact with core LPS, we speculate that *Y. pestis* may hijack APCs, through the core LPS-SIGNR1 interaction, to reach the lymph nodes, utilizing a similar mechanism as demonstrated in the HIV-hDC-SIGN interaction. The knowledge acquired from this study may allow us to develop novel strategies to combat this bacterial pathogen by blocking the interaction between *Y. pestis* and host receptors.

## Materials and Methods

### Ethics Statement

All animal procedures were carried out in strict accordance with the guidelines of Institutional Animal Care and Use Committees (IACUCs) and Institutional Review Board (IRB) of Tongji Hospital, HUST. The handling of the mice and all experimental procedures were specifically approved for this study by the Medical Ethics Committee of Tongji Hospital and were conducted in accordance with the institutional guidelines (IRB ID: TJ-A20141220).

### Bacterial Strains (Table 1)

*E. coli* K12 strain CS180 contains core LPS but lacks O-antigen (75). CS1861 is the strain of CS180 harboring pSS37, a plasmid containing all the genes necessary for the expression of the *Shigella dysenteriae* 1 O-antigen (50, 51, 75). A deep rough isogenic mutant CS2429 (*waaC*), lacking both O-antigen and most of core (50, 51, 75), was used to assess the role of LPS in bacterial-macrophage interactions. *E. coli* strains were cultured on Luria-Bertani medium (LB) supplemented with 1.5% agar at 37°C overnight.

*Yersinia pseudotuberculosis* (Y1) is a serotype O:1a strain, lacking the virulence plasmid (pYV) and expression of Ail protein. The strain was obtained from the CDC and used as a control strain for invasion (43), since this bacterium invades almost all epithelial cell lines via an invasin-integrin interaction (76).

The *Y. pestis* strain 1418 used in this study is originated from KIM5 (KIM-D27), whose *pgm* (pigmentation) 104 kb locus has been deleted (47, 48), and it is therefore classified as an avirulent and non-selected strain. KIM10-Δ*ail* is a derivative of KIM5, in which the *ail* gene has been deleted and its pPCP1 plasmid was also cured (48). The KIM10-Δ*ail* used in this study is derivative of KIM5 and was also pYV plasmid-cured strain, selected using a combination of magnesium oxalate and Congo red selection methods (77). KIM10-Δ*ail*-O^+^ that expresses an O-antigen from *Y. enterocolitica* serotype O:3 (61) is an isogenic derivative of KIM10-Δ*ail*. KIM10-Δ*ail*-Core^**−**^ is also an isogenic derivative of KIM10-Δ*ail*, in which the outer core LPS has been deleted as described in the construction procedures shown below. *Y. pestis* core LPS mutants 5147, 5149, 5150, 5151, and 5188 were generously provided by Dr. Skurnik (46). Strains were cultured on GC based-plates (Difco, Sparks, MD) supplemented with 1% hemoglobin (USB Co., Cleveland, OH).

### Construction of *GmhA*-Deletion Mutant by Allelic Exchange

For construction of *gmhA-*deletion KIM10-Δ*ail*, we followed the procedures described by Dr. Darby (78). The inner core structure contains KDO linked to lipid A followed by heptoses to which the outer core hexoses attach (Figure 1). Phosphoheptose isomerase, encoded by *gmhA*, catalyzes the first step in the GDP-heptose biosynthesis pathway (78). *Y. pestis* KIM6 *gmhA* mutants are deep rough mutants as they do not make GDP-heptose that results in truncation of the outer core of LPS (78). To construct a deep rough mutant derivative of KIM10-Δ*ail*, the *gmhA* allelic exchange plasmid pCBD41 was mobilized from *E. coli* SM10λ*pir/*pCBD41 into KIM10-Δ*ail*. pCBD41 (a kind gift of Dr. Greg Darby) contains two *gmhA-*flanking 900 bp PCR-amplified fragments cloned into suicide vector pCVD442 (78). KIM10-Δ*ail* transconjugants were selected on *Yersinia* selective agar (Difco) containing chloramphenicol and ampicillin. As the suicide vector replication requires the *pir* gene, which does not present in *Yersinia* spp., the recovered KIM10-Δ*ail* transconjugants should contain the plasmid cointegrated at the *gmhA* chromosomal locus. The deletion of *gmhA* was confirmed by PCR assays using the primers; GMHF 5′-GCTTGGATCCCATAATGAAGCTCCTGAGATGTAG and GMHR 5′-AGTGGGTCGACACAGAAGATTGAGGTGATCAAC.

### Construction of *Y. pestis* That Expresses O-Antigen

The expression of O-antigen by *Y. pestis* was lost during evolution from the ancestor, *Y. pseudotuberculosis*. To express the O-antigen, *Y. pestis* strain 1418 was transformed with plasmid pAY100.1 that carries all the necessary genes for the expression of the O-ag of *Y. enterocolitica* serotype O:3 (60, 61). The expression of O-antigen, coded by pAY100.1 plasmid, is not influenced by growth temperature (25–27).

### Cell Lines (Table 1)

Two mouse macrophage cell lines were purchased from ATCC. The CRL-2455 is an alveolar macrophage cell line. J774A.1 was selected, since this cell line shows its ability to phagocytose *Y. pestis* when grown at 26°C (54, 55).

Mouse C-type lectin tranfectants, CHO-mDC-SIGN, CHO-SIGNR1, CHO-SIGNR3, CHO-mDEC-205 (CD205), and CHO-mLangerin (CD207) were generated by transfecting CHO cells (purchased from purchased from the Type Culture Collection of the Chinese Academy of Sciences, Shanghai, China) with mouse corresponding C-type lectin cDNA followed by selection for stable surface expression as originally described (41). CHO-Neo is the control cell line, which expresses the neomycin resistance gene only.

HeLa-DC-SIGN cells were generated by transfecting HeLa cells (purchased from ATCC, USA) with human DC-SIGN cDNA followed by selection for stable surface DC-SIGN expression as originally described (79, 80). The cell lines were recently used for identification of core LPS from several Gram-negative bacteria as ligand for DC-SIGN receptor (22–24).

### Mice

C57BL/6J and BALB/cJ were purchased from Wuhan University Animal Center. SIGNR1 KO mice were kindly provided by The Consortium for Functional Glycomics (CFG, http://www.functionalglycomics.org) and bred in the animal facility of Tongji Hospital. Mice were housed in direct accordance with guidelines drafted by the Animal Care Committees of Tongji Hospital.

### Biology Reagents

Anti-mouse SIGNR1 antibody was purchased from Pharmingen (San Diego, CA). YTH71.3, a rat antibody which recognizes CEACAM1 (CD66a), CEACAM6 (CD66c), and CEACAM3 (CD66d), was purchased from Roche (Indianapolis, IN).

Oligosaccharides: β-D-Gal-(1 → 6)-D-GlcNAc {2-Acetamido-2-deoxy-6-O-(β-D-galactopyranosyl)-D-glucopyranose, A7916}, β-D-Gal-(1 → 4)-D-GlcNAc {N-Acetyl-D-lactosamine A7791}, β-D-Gal-(1 → 3)-D-GalNAc {Galacto-N-biose, A0167}, β-D-GlcNAc-(1 → 3)-β-D-Gal-1 → OMe {(Methyl 3-O-(N-acetyl-β-D-glucosaminyl)-β-D-galactopyranoside, M0775}, α-NeuNAc-(2 → 3)-β-D-Gal-(1 → 4)(α-L-Fuc-)-D-GlcNAc {3'-Sialyl-Lewis-X tetrasaccharide, S1782} and β-D-GlcNAc-(1 → 6)-β-D-Gal-(1 → 4)-D-Glc {β 6'-GlcNAc-lactose, A8297)}, mannan and lactoferrin were purchased from Sigma-Aldrich (St. Louis, MO). Mannan is a ligand antagonist of human mannose receptors. For purpose of easy labeling, the product numbers of each oligosaccharide from Sigma are also included. The background information of each product is listed in Sigma-Aldrich catalog.

Mermaid is a DC-SIGN-like molecule expressed by the marine nematode *Laxus oneistus*. The carbohydrate recognition domain of Mermaid shares both structural and functional similarity with that of DC-SIGN as described (56). A recombinant form of Mermaid (His-Mermaid) was expressed and purified as described (56).

### Sequencing of Six O-Antigen Synthesis Genes

*wbyL, fcl, gmd, wzy, wbyI*, and *ddhB* of 39 *Y. pseudotuberculosis* strains and 8 *Y. pestis* strains were sequenced. First, these genes were copied by Polymerase Chain Reaction using high-fidelity DNA polymerase (PCR SuperMix, Transgene Biotech, Beijing). And then the products were sent to perform bidirectional Sanger sequencing. Primers for these O-antigen synthesis genes; wbyL Forward 5′- GTCGGCATTGCTCATTCTATTG- 3′, wbyL Reverse 5′- TCACTGGTTAATCGAACATCCC- 3′, fcl Forward 5′- TGCTGAAATGGTCGCTAGTG-3′, fcl Reverse 5′- AGAGTCGCCATATCCAAATAGC-3′, gmd Forward 5′- AGGTGATGCCGCTATATTAGTG-3′, gmd Reverse 5′- GAGGTCAAGTTCAGTACGATCC-3′, wzy-1 Forward 5′- TCGACTACCTTCTCATTCTTGG-3′, wzy-1 Reverse 5′- TCACGACGAAGAGCCTTTATAG-3′, wzy-2 Forward 5′- GGCCTCTTGTACCAAACTTC-3′, wzy-2 Reverse 5′- TCCGAGAAATAGACAGTTACCC-3′, wbyI Forward 5′- TGTGTCAAGTTAGTCGGATATG-3′, wbyI Reverse 5′- CTTGCGAAGACCATTTCATTAG-3′, ddhB Forward 5′- GGCAGGGCACCTTGGAAG-3′, and ddhB Reverse 5′- CCAGCTCAGCAATCTGTTGAC-3′. Sequencing data was analyzed by BioNumerics Software Version 7 and heatmap was made by R software.

### Isolation of Mouse Peritoneal Macrophages

The peritoneal macrophages were selected as our primary cells. After the 6- to 8-week-old female mice were euthanized, intact abdomen was exposed, cleaned with 70% ethanol and opened. Five milliliter of RPMI was injected into intraperitoneal cavity. Mouse abdomen was gently massaged for 3 min and then the lavage fluid was collected. The suspension containing the macrophages was seeded in flasks and placed in a CO_2_ incubator for 2 h. The cell layers were washed 3 times to remove non-adherent cells. Macrophages were then removed from the plastic surface by incubating with citrate saline and re-seeded for interaction assays or stained with antibodies to check the expression level of receptors.

### Adherence and Phagocytosis Assays

The assays for adherence and phagocytosis have been described previously (81, 82). Briefly, host cells (CHO, HeLa, and macrophages) were plated in 24 or 96-well plates. Cells were suspended in RPMI with 2% FCS at a concentration of 4 × 10^5^/ml. One half ml each of these cell suspensions was added to 24-well plates and after addition of 50 μl of bacterial suspensions at a concentration of 1 × 10^7^ colony forming units (CFU)/ml, the cells were allowed to incubate for 2.5 h (2 h for alveolar macrophages) at 37°C in the presence of 5% CO_2_. The cell monolayers were then washed 3 times with PBS. The number of associated bacteria (adherent and internalized) per cell was quantified by washing the cells 3 times with RPMI containing 2% FCS and plating the culture after the cells were lysed by 0.5% saponin (Calbiochem Corp., San Diego, CA).

To determine the internalization of bacteria, gentamicin, which kills extracellular bacteria but cannot penetrate host cells, was added into each well to a final concentration of 100 μg/ml, and the cultures were incubated for 60 min. Cells were washed twice to remove the antibiotics. Then, the cells were suspended in PBS containing 0.5% saponin, diluted and plated on LB and GC as well as *Y. pestis* plates. The level of internalization of bacteria in these host cells was calculated by determining the CFU recovered from lysed cells.

For the inhibition assay, reagents were added 20 min prior to the addition of bacteria at the following concentrations: anti-SIGNR1 antibody, 5 μg/ml; mannan, 500 μg/ml; DC-SIGN-like protein (Mermaid), 10 μg/ml and anti-CD66, 5 μg/ml. The concentrations used were based on our preliminary data, and were selected based on the fact that at these concentrations, there was no influence on the survival of bacteria and HeLa cells, or the interaction between pEXI and HeLa-CEACAM3 (22–24, 82, 83).

### Determination of Phagocytosis by Flow Cytometry

The following method was used to supplement the survival-based phagocytosis assay described previously (23). Briefly, bacteria were suspended in RPMI medium containing 5- and 6-carboxyfluorescein diacetate, succinimidyl ester (CFDA-SE; Molecular Probes, Eugene. OR) for 40 min and washed twice with RPMI to remove the excess dye. Labeled bacteria were added to macrophage cultures for 2 h. Cell cultures were washed twice to remove unbound bacteria. Macrophages plus associated bacteria were fixed with 2% paraformaldehyde. Before flow cytometry, a 1:10 dilution of Trypan blue (0.4%, Sigma, St. Louis, MO) was added to the fixed cell cultures and the mixture was incubated at ambient temperature for 10 min (23) to quench the fluorescence from extracellular labeled bacteria. Trypan blue blocks fluorescence but cannot penetrate host cells, therefore, fluorescence from internalized bacteria will not be influenced by addition of Trypan blue. The rate of bacterial internalization was determined by comparing the intensity fluorescence-positive macrophages with various bacteria. The higher of the fluorescence-intensity shows, the more of bacteria are phagocytosed by macrophages.

### *In vivo* Phagocytosis Assays

One milliliter of bacterial suspensions (OD = 0.1) were injected into 6- to 8-week-old female mouse intraperitoneal cavity. Mouse abdomen was gently massaged for 1 min. After 1.5 h, mice were euthanized and another 4 ml of RPMI with 2% FCS were immediately injected into each mouse peritoneal cavity and the abdomen was gently massaged for another 1 min. The intraperitoneal fluids or exudates were collected, and the numbers of cells were counted for each collection of intraperitoneal lavage. 1 × 10^6^ was seeded onto each well of 24-well plates, containing RPMI with 2% FCS and gentamycin at concentration of 100 μg/ml, and were then incubated for 1.5 h to allow the macrophages adhere to plates and kill the extracellular bacteria. Each well was washed three times with RPMI with 2% FCS to remove non-adhered cells and lysed with saponin, followed the same procedures as *in vitro* phagocytosis assays.

### Animal Challenging for Dissemination and Infection

Six- to eight-week-old female C57BL/6J and SIGNR1 KO mice were used in the following experiments. The dissemination rate was defined as the transport of *Y. pestis* to LN, spleen, and liver. The infectivity was defined as the mortality after inoculations of pathogens.

The protocol follows a similar assay we previously developed (26, 27). *Y. pestis* were cultured at 26°C to avoid the expression of OPM capsule and then suspended in PBS at a concentration of OD_600_ = 1.5 in PBS. Hundred microliters of the *Yersinia* suspension was injected in hind paws of mice. It should be noted 30 min before inoculation, mice were injected with ampicillin at a final concentration of 50 μg/g of mouse body weight to maintain the plasmid-based expression of O-antigen. (1) For CFU determination, the mice were euthanized and the inguinal lymph nodes were isolated 24 h post-injection. The isolated inguinal lymph nodes were then homogenized and lysed with 0.5% Triton X-100 to release the bacteria prior to plating onto agar plates containing ampicillin. The total isolated CFU of inguinal lymph nodes per mouse were defined as the dissemination rate. (2) For bioluminescence imaging, C57BL/6J mice were inoculated with *Y. pestis* 1418 and *Y. pestis* 1418-O^+^ transformed with the plasmid pXEN-18 which expresses the *lux* genes. The bioluminescence signal was detected by Night OWL II LB983 imaging system (Berthold Technologies, Bad Wildbad, Germany).

### Statistical Analyses

All statistical analyses were completed using Prism software, Version 6 (Graph Pad, San Diego, CA, USA). Statistical significance was assessed using Student's unpaired *t*-test. Survival assay was analyzed by log-rank test.

## Author Contributions

TC, CP, YK, AA, RY, JK, and MS contributed conception and design of the study. KY, YXH, YPH, and YJC performed the assays. YXH and YPH performed the statistical analysis. KY and YXH wrote the first draft of the manuscript. All authors contributed to manuscript revision, read and approved the submitted version.

### Conflict of Interest Statement

The authors declare that the research was conducted in the absence of any commercial or financial relationships that could be construed as a potential conflict of interest.
